# Outcomes of percutaneous coronary intervention for chronic total occlusions in the elderly: A systematic review and meta‐analysis

**DOI:** 10.1002/clc.23524

**Published:** 2020-12-17

**Authors:** Chenmin Cui, Zhichao Sheng

**Affiliations:** ^1^ Department of Nephrology Huzhou Hospital of Traditional Chinese Medicine Affiliated Zhejiang University of Traditional Chinese Medicine Huzhou China; ^2^ Department of Cardiovascular Medicine Xinchang Hospital of Traditional Chinese Medicine Shaoxing China

**Keywords:** chronic total occlusions, complications, coronary artery disease, mortality, percutaneous coronary intervention, survival

## Abstract

**Objective:**

This study aimed to compare outcomes of percutaneous coronary intervention (PCI) for chronic total occlusions (CTO) in the elderly (≥75 years) versus nonelderly and assess the impact of successful CTO‐PCI in the elderly.

**Methods:**

PubMed, Embase, ScienceDirect, CENTRAL, and Google Scholar databases were searched up to October 1, 2020. Mortality rates and major adverse cardiac events (MACE) were compared between elderly and nonelderly patients and successful versus failed CTO‐PCI in the elderly.

**Results:**

Eight studies were included. Meta‐analysis indicated no statistically significant difference in the risk of in‐hospital mortality (RR: 1.97 95% CI: 0.78, 4.96 I^2^ = 0% p = .15) but higher tendency of in‐hospital MACE (RR: 2.30 95% CI: 0.99, 5.35 I^2^ = 49% p = .05) in the elderly group. Risk of long‐term mortality (RR: 3.79 95% CI: 2.84, 5.04 I^2^ = 41% p < .00001) and long‐term MACE (RR: 1.53 95% CI: 1.14, 2.04 I^2^ = 80% p = .004) were significantly increased in the elderly versus nonelderly. Elderly patients had a significantly reduced odds of successful PCI as compared to nonelderly patients (OR: 0.63 95% CI: 0.54, 0.73 I^2^ = 1% p < .00001). Successful CTO‐PCI was associated with reduction in long‐term mortality (HR: 0.51 95% CI: 0.34, 0.77 I^2^ = 27% p = .001) and MACE (HR: 0.60 95% CI: 0.37, 0.97 I^2^ = 53% p = .04) as compared to failed PCI in elderly.

**Conclusions:**

Elderly patients may have a tendency of higher in‐hospital MACE with significantly increased long‐term mortality and MACE after CTO‐PCI. The success of PCI is significantly lower in the elderly. In elderly patients with successful PCI, the risk of long‐term mortality and MACE is significantly reduced.

## 1 INTRODUCTION

Over the past several years, owing to improvements in public health programs and the availability of high‐quality medical care, there has been an increase in life expectancy. The cohort of the elderly population is increasing with a corresponding increase in the prevalence of the cardiac disease, especially coronary artery disease (CAD).^1^ According to angiographic data, chronic total occlusions (CTO) constitute around 18.4% of lesions in patients with significant CAD.^2^ The prevalence of CTO is especially high in the elderly and the presence of other comorbidities like peripheral artery disease, stroke, hypertension, and diabetes significantly complicates the management of these lesions in older adults.^3^

Historical data indicates that coronary artery bypass graft (CABG) surgery or medical therapy were commonly used strategies for CTO.^4^ However, over the years with improvement in percutaneous coronary intervention (PCI) techniques like availability of sophisticated guidewire, use of retrograde approach, and antegrade dissection and reentry methods have significantly improved the success of CTO‐PCI. An increasing number of patients are now undergoing this percutaneous procedure for CTO.^5^ Evidence suggests that successful PCI for CTO is associated with improved survival as well as the reduction of adverse cardiac events.^6^, ^7^ Kirschbaum et al.^8^ have demonstrated that revascularization of CTO with PCI leads to improvement in left ventricular remodeling and ejection fraction, which is observed up to 3 years post‐PCI.

Despite evidence suggesting favorable outcomes with PCI for CTO, the procedure is infrequently performed in the elderly population. The reluctance stems from the fact that the disease is more complex in older patients and evidence from non‐CTO PCI studies indicating higher complications and mortality in the elderly.^9^ The cohort of older patients is frequently excluded from clinical trials and registries and there is a dearth of evidence on the outcomes of CTO‐PCI in the elderly. To make informed clinical decisions, there is a need to answer the following questions: (1) Is there a difference in clinical outcomes following CTO‐PCI in the elderly versus nonelderly patients and (2) Does the success of CTO‐PCI in elderly patients results in improved clinical outcomes as compared to failed procedures? To the best of our knowledge, only one systematic review^10^ has attempted to analyze evidence on the subject in question and no meta‐analysis has been conducted in the literature to present pooled evidence on the outcomes of CTO‐PCI in the elderly. Thus, our study aimed to conduct a systematic literature search and pool data from relevant studies to compare outcomes of CTO‐PCI in the elderly versus nonelderly and assess the impact of successful CTO‐PCI on clinical outcomes in the elderly.

## 2 METHODS

### 2.1 Inclusion criteria

The review is conducted as per the guidelines of the PRISMA statement (preferred reporting items for systematic reviews and meta‐analyses).^11^ We included all studies conducted on elderly patients (≥75 years of age) undergoing PCI for CTO. Studies were included provided they fulfilled one of the following criteria:

1. Studies were to compare outcomes of elderly patients with nonelderly patients (<75 years of age). Outcomes reported were to be mortality and/or major adverse cardiac events (MACE).

OR

2. Studies were to compare outcomes of successful and failed PCI for CTO in elderly patients. Outcomes of interest were mortality and/or MACE.

No restriction was placed on the study design, sample size, language of publication, or date of publication. The following were the exclusion criteria for the review: 1. Studies comparing outcomes of PCI versus medical therapy only. 2. Studies comparing outcomes of PCI versus CABG only. 3. Studies not segregating data based on elderly and nonelderly subjects. 3. Studies using any other definition of the elderly population (i.e., ≥60 or ≥ 65 years). 4. Studies not reporting relevant data. 5. Review articles and unpublished studies were also excluded.

### 2.2 Search strategy

An electronic search was conducted by two reviewers, independent of each other, for the following databases: PubMed, Embase, ScienceDirect, CENTRAL, and Google Scholar. The time limit was from the inception of databases to October 1, 2020. The terms used for the literature search included: “percutaneous coronary intervention,” “chronic total occlusion,” “elderly,” “older adults,” “geriatric,” and “age.” Search terms were used in different combinations to find relevant articles. After the deduplication of articles, the search records were analyzed by their titles and abstracts separately by the two reviewers. Articles matching the inclusion criteria were identified and full texts of these were extracted. Individual studies were then assessed for final inclusion in the study. Any disagreements were resolved by discussion. After completion of the search and identification of included studies, the bibliography of included articles was hand searched for any other potential article.

### 2.3 Data extraction and quality of included studies

The following data were extracted from the included studies: names of first authors, publication year, study type and location, study groups, sample size, demographic details of the sample, medical history of the sample (hypertension, diabetes, hyperlipidemia, chronic heart failure, chronic kidney disease, prior MI, stroke, CABG, or PCI), the success of PCI, CTO location, contrast volume use, procedural time, study outcomes, and follow‐up time.

For the first part of the review, mortality and MACE were compared following CTO‐PCI in elderly versus nonelderly. We also performed a separate analysis comparing major bleeding, cardiac tamponade, emergent CABG, MI, and cerebrovascular accident (CVA) between the elderly and nonelderly groups. Finally, the success rates of PCI were compared between the elderly and nonelderly groups. For the second part of the review, we compared mortality and MACE between successful PCI versus failed PCI for CTO in the elderly.

Since only observational studies were included in the review, the risk of a bias assessment tool for nonrandomized studies (RoBANS) was used to assess the quality of included studies.^12^ Studies were assessed for the selection of participants, confounding variables, intervention measurements, blinding of outcome assessment, incomplete outcome data, and selective outcome reporting. Two reviewers independently assessed each study. The study was judged to have a “high,” “unclear,” or “low” risk of bias for each domain. Any disagreements were resolved by discussion.

### 2.4 Statistical analysis

“Review Manager” (RevMan, version 5.3; Nordic Cochrane Centre [Cochrane Collaboration], Copenhagen, Denmark; 2014) was used for the meta‐analysis. Using a random‐effects model, all categorical adverse outcomes were summarized using risk ratios (RR) with 95% confidence intervals (CI). The success of PCI between elderly and nonelderly was compared using odds ratios (OR). We also extracted data on hazard ratio (HR) for mortality or MACE if reported by the included studies. The generic inverse variance model of the meta‐analysis software was used to pool the HR. Meta‐analysis was conducted only if at least three studies reported the same outcome. Heterogeneity was assessed using the I^2^ statistic. I^2^ values of 25–50% represented low, values of 50–75% medium, and more than 75% represented substantial heterogeneity. As less than 10 studies were included in the meta‐analysis, funnel plots were not used to assess publication bias.

## 3 RESULTS

**FIGURE 1:**
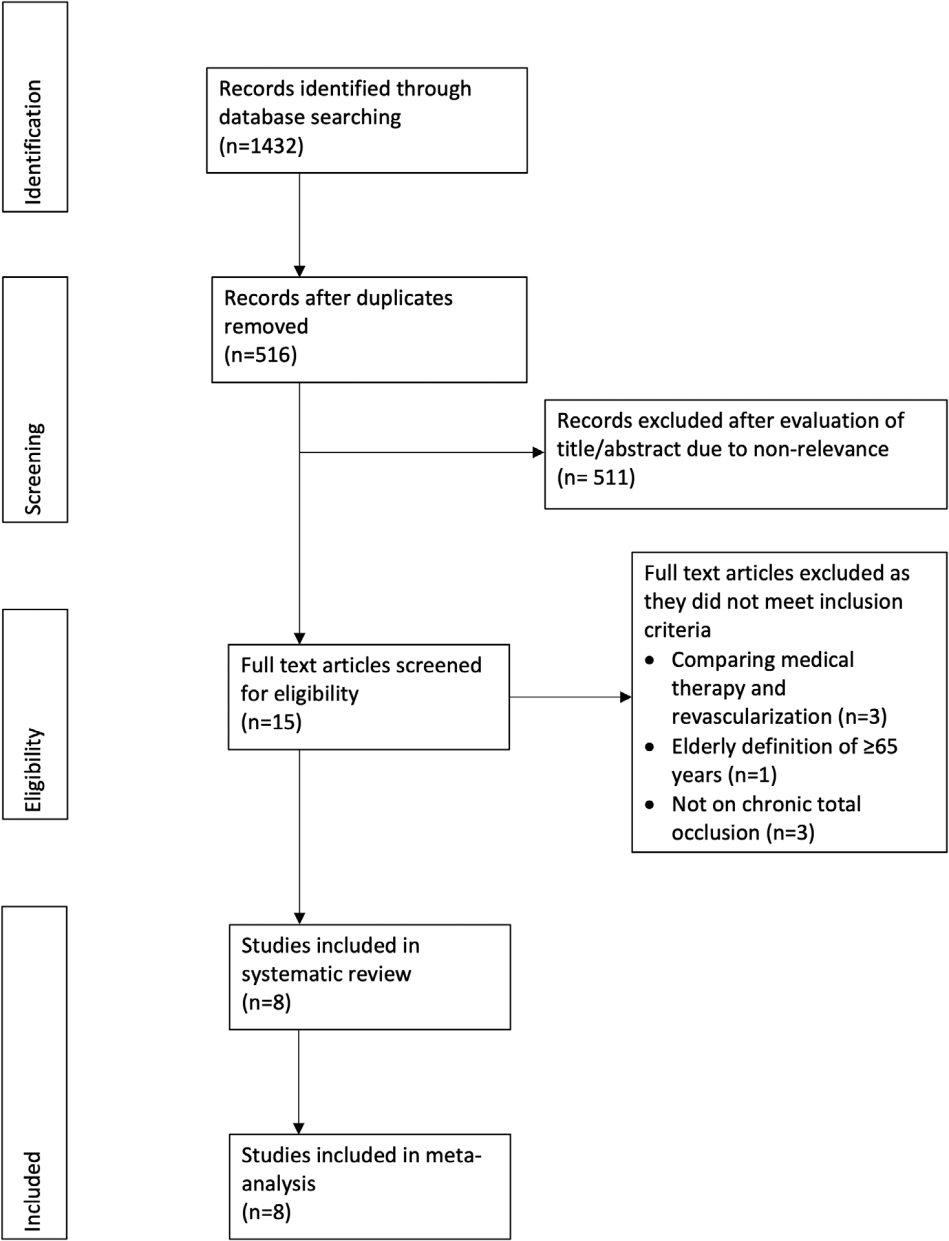
Study flow chart

The PRISMA flowchart of the review is presented in Figure 1. A total of eight studies^13^, ^14^, ^15^, ^16^, ^17^, ^18^, ^19^, ^20^ fulfilled the inclusion criteria. Seven studies^14^, ^15^, ^16^, ^17^, ^18^, ^19^, ^20^ compared CTO‐PCI outcomes between elderly and nonelderly subjects of which five studies^13^, ^15^, ^18^, ^19^, ^20^ also compared outcomes of successful versus failed CTO‐PCI in the elderly. One study^13^ compared only successful and failed CTO‐PCI in the elderly without a nonelderly comparison group. Table 1 presents the baseline details of the included studies with data on demographics and baseline medical history of the sample. Except for two,^18^, ^20^ all were retrospective studies. There were statistically significant differences reported in the baseline variables of the two groups (elderly and nonelderly) by the included studies. Follow‐up ranged from 20 months to 5 years in the included studies. Details of the success of the PCI procedure and other procedural variables are presented in Table 2. The included studies reported a varying percentage of CTO‐PCI success in the elderly ranging from 63.9 to 84%. Quality assessment of included studies is presented in Supplementary Table S1.

**TABLE 1:** Characteristics of included studies

Author/Year	Study type	Location	Groups	Sample size	Mean Age	Male gender (%)	HTN (%)	HLD (%)	DM type 2 (%)	CHF	CKD	Smokers (%)	Prior MI (%)	Prior PCI (%)	Prior CABG (%)	Prior stroke (%)	Follow‐up
Valenti et al.^13^/2019	RT	Italy	≥75	460	80.5 ± 4	76	69	51	28	NR	17	NR	53	37	16	NR	5 years
Su et al.^14^/2019	RT	China	<75 ≥75	178 68	62.7 ± 8.4 79.4 ± 3.2	**76.4** **55.8**	74.1 85.2	**16.8** **5.8**	37.6 33.8	NR	**3.93** **11.7**	**31.4** **17.6**	23.6 16.1	39.8 42.6	NR	**20.2** **44.1**	NR
Toma et al.^16^/2017	RT	Germany	<75 ≥75	1593 409	61.5 ± 9 79.5 ± 4	**14.1** **26.4**	**80.1** **90.7**	**85.9** **83.1**	28.9 31.5	16.4 21	**10.8** **5.4**	**23.7** **5.9**	24.5 25.2	15.1 16.9	**13.9** **17.4**	NR	3 years
Karatasakis et al.^17^/2017	RT	USA	<75 ≥75	1391 253	63.1 ± 6 80.1 ± 3	87.5 75.9	89.4 92.9	**94** **98**	45.5 41.1	28.5 30.8	NR	**28** **11.9**	44.5 45.1	63.5 64	**35** **43.1**	10.5 15.8	NR
Zhang et al.^15^/2017	RT	China	<75 ≥75	325 120	63 ± NR 78 ± NR	75.7 67.5	65.8 72.5	NR	34.2 33.3	16 20	NR	**61.5** **40.8**	22.2 24.2	NR	NR	NR	3 years
Andre et al.^18^/2016	PT	France	<75 ≥75	263 93	60.6 ± 9 80.5 ± 4	87.8 73.1	**51** **75.3**	**75.7** **63.4**	32.3 39.8	100 100	**19.4** **41.9**	**69.2** **36.6**	51 53.8	44.1 47.3	**5.7** **18.3**	7.2 9.7	20 months
Tanaka et al.^19^/2013	RT	Japan	<75 ≥75	217 67	63.1 ± 9 78.5 ± 3	88.9 65.7	64.1 68.7	65.4 67.2	40.6 34.3	NR	6.9 1.5	23.5 9	17.5 17.9	NR	NR	NR	3 years
Hoebers et al.^20^/2013	PT	USA, Italy, South Korea	<75 ≥75	1578 213	59.1 ± 9 79 ± 3	**87.3** **77.9**	59.1 64.8	**65.4** **56.3**	23.4 17.4	NR	**2.9** **7.4**	27.3 19.7	**49.1** **54**	NR	15.1 22.1	NR	5 years

*Note:* Figures in bold indicate statistical significant differences between the study groups for the variable.

Abbreviations: CABG, coronary artery bypass grafting; CHF, congestive heart failure; CKD, chronic kidney disease; DM, diabetes mellitus; HLD, hyperlipidaemia; HTN, hypertension; MI, myocardial infarction; NR, not reported; PCI, percutaneous coronary intervention.

**TABLE 2:** Success of PCI and angiographic details of included studies

Author/Year	Groups	Successful procedures	CTO location			Multivessel disease	Mean contrast volume (ml)	Mean procedural time (mins)
			LAD	LCX	RCA			
Valenti et al.^13^/2019	≥75	333 (72.4%)	114 (34%)	70 (21%)	133 (40%)	403 (88%)	300 (200–400)*	NR
Su et al.^14^/2019	<75 ≥75	151 (84.8%) 50 (73.5%)	80 (44.9%) 36 (52.9%)	39 (21.9%) 7 (10.2%)	59 (33.1%) 25 (36.7%)	NR	**242 ± 62.9** **182.8 ± 69**	**130.4 ± 46.3** **112.1 ± 42.8**
Toma et al.^16^/2017	<75 ≥75	1355 (85%) 307 (75%)	431 (27%) 125 (30.6%)	765 (48%) 166 (40.6%)	388 (24.4%) 112 (27.4%)	**1279 (80.3%)** **355 (86.8%)**	321 ± 157 315 ± 153	103 ± 55 100 ± 54
Karatasakis et al.^17^/2017	<75 ≥75	1231 (88.5%) 213 (84%)	327 (23.5%) 71 (28%)	313 (22.5%) 45 (18%)	765 (55%) 139 (55%)	NR	275 (200–375)^a^ 250 (200–350)	132 (83–202)^a^ 136 (91–201)
Zhang et al.^15^/2017	<75 ≥75	105 (82.7%) 23 (69.7%)	99 (30.5%) 35 (29.2%)	116 (35.7%) 46 (38.3%)	163 (50.2%) 54 (45%)	50 (15.3%) 15 (12.5%)	NR	NR
Andre et al.^18^/2016	<75 ≥75	226 (78%) 75 (74.3%)	NR	78 (29.7%) 29 (31.2%)	152 (57.8%) 42 (45.2%)	NR	NR	NR
Tanaka et al.^19^/2013	<75 ≥75	189 (79%) 57 (77%)	68 (31.3%) 25 (37.3%)	59 (27.2%) 17 (25.4%)	111 (51.2%) 32 (47.8%)	118 (54%) 32 (48%)	176 ± 80 164 ± 79	NR
Hoebers et al.^20^/2013	<75 ≥75	1090 (69%) 136 (63.9%)	541 (34.3) 77 (36.2%)	369 (23.4%) 38 (17.8%)	31 (42%) 97 (42%)	**1057 (67%)** **166 (77.7%)**	**482 ± 226** **404 ± 197**	NR

*Note:* Figures in bold indicate statistical significant differences between the study groups for the variable.

Abbreviations: LAD, Left anterior descending; LCX, Left circumflex; NR, Not reported; RCA, Right coronary artery.

^a^ Median (interquartile range).

### 3.1 Elderly versus nonelderly

**FIGURE 2:**
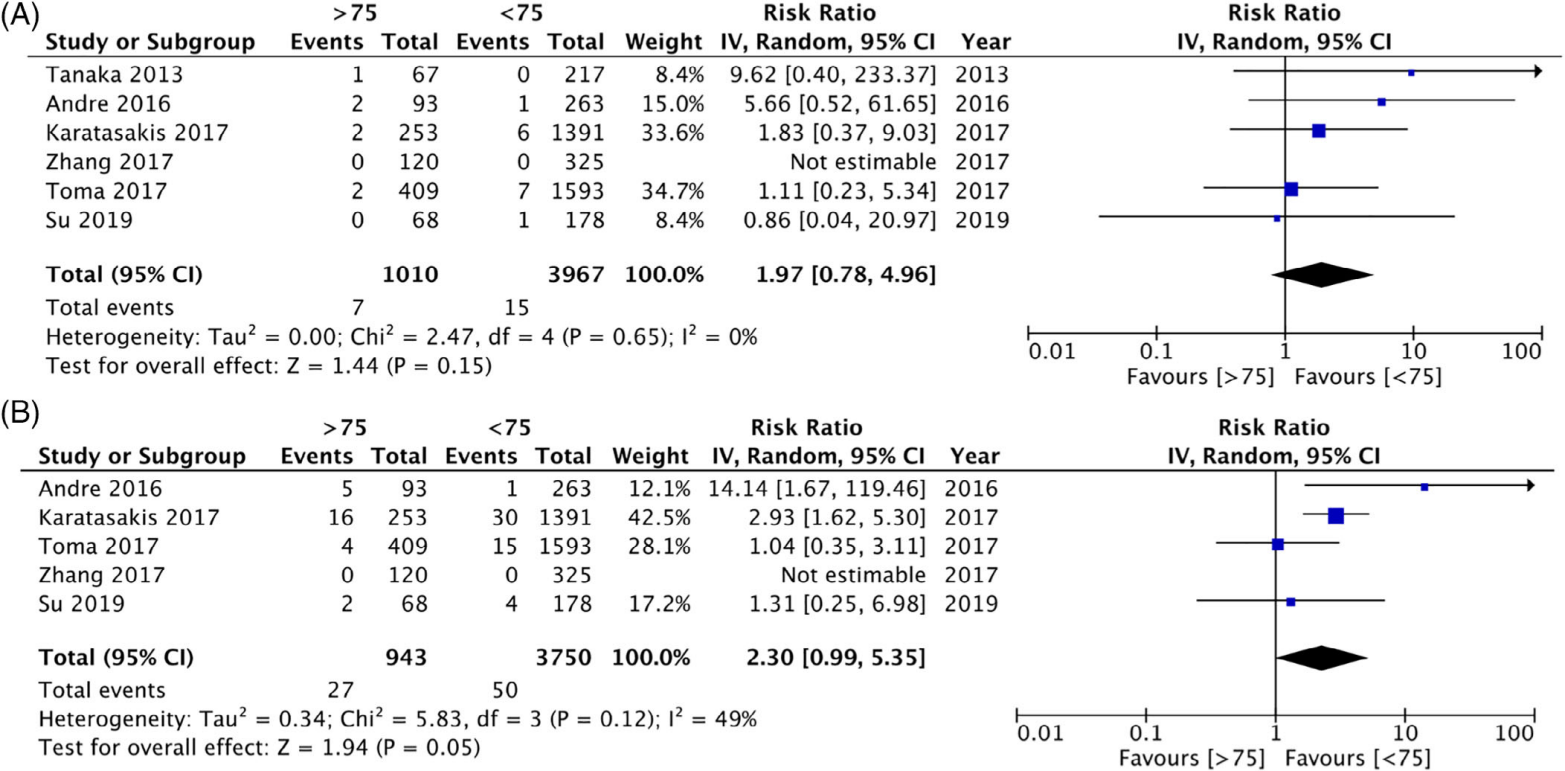
(A) Forest plot of in‐hospital mortality after CTO‐PCI in elderly versus nonelderly. (B) Forest plot of in‐hospital MACE after CTO‐PCI in elderly versus nonelderly. CTO, chronic total occlusions; MACE, major adverse cardiac events; PCI, percutaneous coronary intervention

In‐hospital outcomes (i.e., short term outcomes) and long‐term outcomes were compared separately for this part of the analysis. A meta‐analysis of data from 4977 patients indicated no statistically significant difference in the risk of mortality between elderly and nonelderly patients undergoing CTO‐PCI (RR: 1.97 95% CI: 0.78, 4.96 I^2^ = 0% p = .15) (Figure 2A). Pooled analysis of data from 4693 patients revealed a tendency for increased risk of MACE in the elderly, but the results were not statistically significant (RR: 2.30 95% CI: 0.99, 5.35 I^2^ = 49% p = .05) (Figure 2B). On further analysis of specific adverse events, the risk of major bleeding (RR: 3.17 95% CI: 1.19, 11.46 I^2^ = 54% p = .02) (Supplementary Figure S1), emergent CABG (RR: 6.44 95% CI: 1.05, 39.36 I^2^ = 0% p = .04) (Supplementary Figure S2) and MI (RR: 2.43 95% CI: 1.18, 5.04 I^2^ = 0% p = 0.02) (Supplementary Figure S3) was significantly increased in the elderly as compared to nonelderly patients. No statistically significant differences were noted in the risk of cardiac tamponade (RR: 2.01 95% CI: 0.59, 6.82 I^2^ 44% p = .26) (Supplementary Figure S4) and CVA (RR: 1.89 95% CI: 0.29, 12.16 I^2^ = 35% p = .50) (Supplementary Figure S5) between the two groups.

**FIGURE 3:**
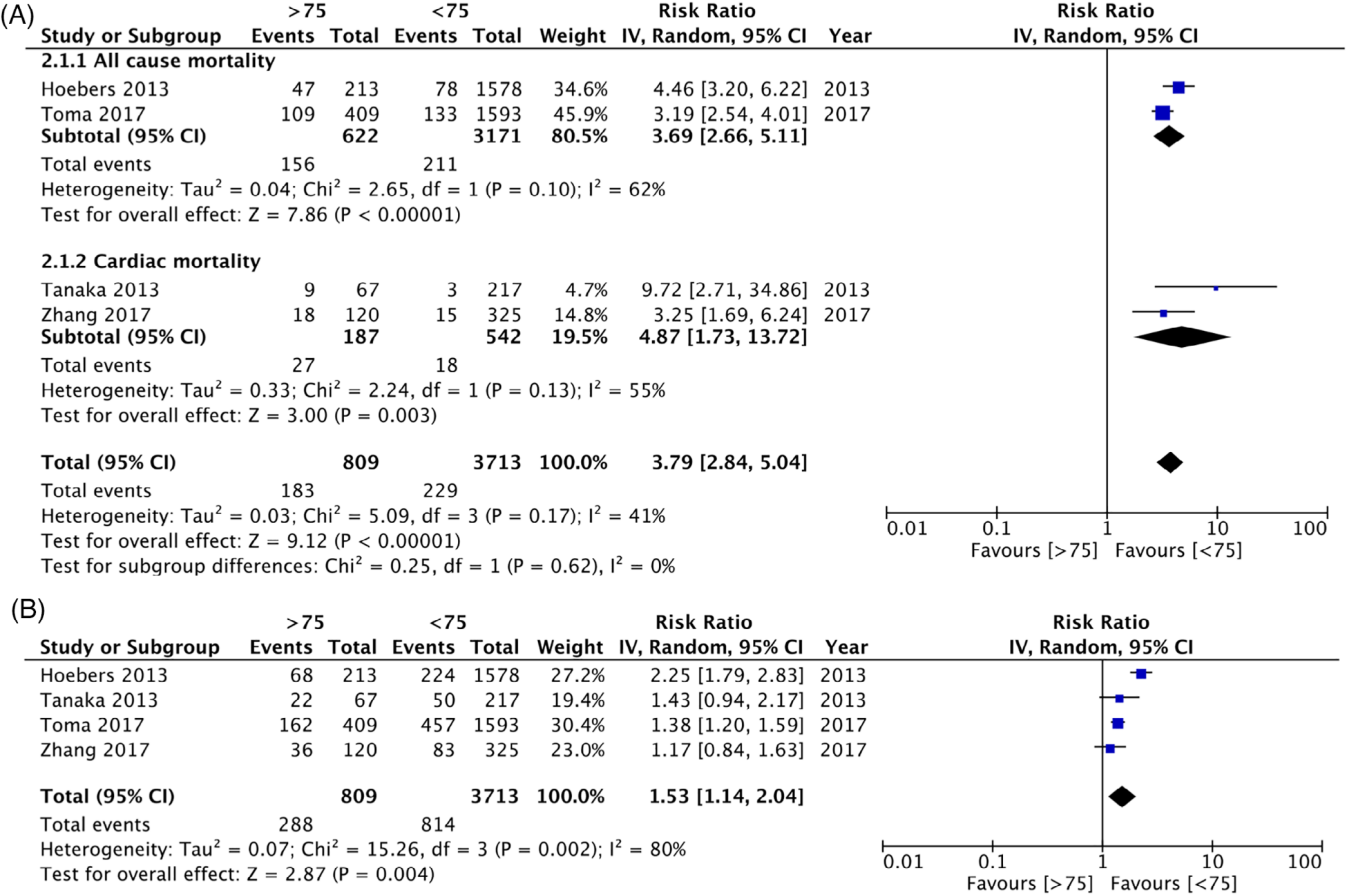
(A) Forest plot of long‐term mortality after CTO‐PCI in elderly versus nonelderly. (B) Forest plot of long‐term MACE after CTO‐PCI in elderly versus nonelderly. CTO, chronic total occlusions; MACE, major adverse cardiac events; PCI, percutaneous coronary intervention

Four studies reported data on long‐term mortality, albeit with different follow‐ups. Two reported data on cardiac mortality while two reported all‐cause mortality. Pooled analysis of data of 4522 patients revealed a significantly increased risk of mortality in the elderly as compared to nonelderly (RR: 3.79 95% CI: 2.84, 5.04 I^2^ = 41% p < .00001) (Figure 3A). Results were significant for both all‐cause (RR: 3.69 95% CI: 2.66, 5.11 I^2^ = 62% p < .00001) and cardiac mortality (RR: 4.87 95% CI: 1.73, 13.72 I^2^ = 55% p = .003). A meta‐analysis of four studies reporting data on long‐term MACE indicated a significantly increased risk of MACE in the elderly undergoing CTO‐PCI (RR: 1.53 95% CI: 1.14, 2.04 I^2^ = 80% p = .004) (Figure 3B). On comparison of the success of PCI, elderly patients had a significantly reduced odds of successful PCI as compared to nonelderly patients (OR: 0.63 95% CI: 0.54, 0.73 I^2^ = 1% p < .00001) (Supplementary Figure S6).

### 3.2 Successful versus failed PCI in the elderly

Of the five studies reporting data on long‐term mortality for this analysis, two reported all‐cause mortality while the remaining reported cardiac mortality. A meta‐analysis revealed no statistically significant difference in long‐term mortality between successful and failed PCI groups (RR: 0.77 95% CI: 0.45, 1.30 I^2^ = 39% p = .32) (Supplementary Figure S7). Sub‐group analysis revealed no significant difference for all‐cause mortality (RR: 1.11 95% CI: 0.43, 2.90 I^2^ = 49% p = .83) as well as for cardiac mortality (RR: 0.56 95% CI: 0.24, 1.30 I^2^ = 39% p = .18). On the other hand, pooled analysis of four studies indicated significantly reduced risk of long‐term MACE in patients with successful PCI as compared to failed PCI (RR: 0.63 95% CI: 0.48, 0.83 I^2^ = 0% p = .001) (Supplementary Figure S8).

Data from studies reporting HR for prediction of mortality and MACE with successful versus failed PCI were also extracted. Pooled analysis indicated that successful PCI was associated significantly reduced risk of mortality (HR: 0.51 95% CI: 0.34, 0.77 I^2^ = 27% p = .001) (Supplementary Figure S9) and MACE (HR: 0.60 95% CI: 0.37, 0.97 I^2^ = 53% p = .04) (Supplementary Figure S10).

## 4 DISCUSSION

Our study, which is the first meta‐analysis assessing the outcomes of CTO‐PCI in the elderly, revealed the following important findings. (1) In‐hospital mortality for CTO‐PCI may not be different between elderly and nonelderly, but there is a tendency of higher risk of early MACE in the elderly group. (2) Risk of long‐term mortality and MACE is significantly increased in the elderly following CTO‐PCI. (3) Success of PCI is significantly lower in the elderly versus nonelderly. 4) In elderly patients with successful PCI, the risk of long‐term mortality and MACE is significantly reduced as compared to those with failed CTO‐PCI.

CTO is common in the elderly population and accounts for poor clinical outcomes in this age group. Therefore, the European Society of Cardiology (ESC) and the European Association for Cardio‐Thoracic Surgery (EACTS) recommend that myocardial revascularization in the presence of CTO be considered if there are symptoms or objective evidence of cardiac ischemia in the area of the occluded artery.^21^ This has been translated into clinical practice where the initial management of CTO in elderly patients is usually by medical therapy and in case of persistent angina or evidence of myocardial insufficiency, revascularization is indicated. Evidence comparing revascularization strategies versus medical therapy alone for CTO in the elderly population is limited. Flores‐Umanzor et al.^22^ in a retrospective study of elderly patients (≥75 years) with CTO have compared 53 PCI procedures, 42 CABG procedures with 233 patients receiving only medical therapy. The authors reported that revascularization with either PCI or CABG in the elderly leads to reduced all‐cause and cardiac mortality. However, revascularization for a CTO with PCI or CABG is a complex procedure and many intervention cardiologists are reluctant to perform invasive treatment in elderly patients. Many retrospective observational studies have demonstrated a lack of statistically significant differences in outcomes with either CABG or PCI in elderly patients.^23^, ^24^ However, due to concerns of neurological complications like cognitive decline in the elderly with CABG combined with the improvement of PCI equipment and techniques, PCI for CTO may be the preferred treatment modality in older adults.^25^, ^26^

In this context, an important question that needs to be answered is if CTO‐PCI has similar outcomes in elderly and nonelderly subjects? The concern of poor outcomes in the elderly with CTO arises from the fact that the disease is more extensive in older adults and coronary arteries are more tortuous with heavy calcification of atherosclerotic plaques.^20^ Moreover, the presence of multiple comorbidities in the elderly like hypertension, diabetes, poor cardiopulmonary function, chronic kidney disease, anemia, and so forth can further complicate outcomes. Galassi et al.^27^ in their model to predict technical failure of CTO‐PCI have found that the age of ≥75 years is associated with poorer outcomes. In our meta‐analysis, we found no statistically significant difference between elderly and nonelderly patients for in‐hospital mortality or MACE. However, on closer examination of the forest plot, the lower end of 95% CI was close to 1 and the upper end of 95% CI for in‐hospital mortality and MACE were 4.96 and 5.35 respectively, indicating an upper limit of ~5 fold risk of mortality and MACE in the elderly population. Similar was the case for the risk of cardiac tamponade and CVA, which too were nonsignificant between the two groups but had a high‐upper end of 95% CI (cardiac tamponade: 6.82; CVA: 12.16). The lack of statistical significance for the above variables may be due to the limited number of studies in the analysis. On the contrary, our analysis did find 3.17 times increased risk of major bleeding, 6.44 times increased risk of emergent CABG and 2.43 times increased risk of MI in the elderly undergoing CTO‐PCI. The risk of long‐term mortality and MACE were also significantly higher in the elderly. The results of our study are consistent with reports of non‐CTO PCI in the elderly. Feldman et al^28^ in a cohort of 10 964 patients have demonstrated that age is a strong predictor of in‐hospital mortality and MACE in both elective and emergent PCI in a multivariable analysis model. Chen et al.^9^ in a comparative study of ≥75 and < 75‐year‐old adults reported increased mortality in the older age group. Thomas et al.^29^ in an analysis of 152 373 patients undergoing PCI have reported an increased risk of mortality, contrast‐induced nephropathy, bleeding, CVA, and vascular complications with increasing age.

The success of PCI for CTO in the literature ranges widely from 59 to 87.5%.^30^, ^31^ The success rates for CTO‐PCI are significantly lower as compared to nonoccluded lesions and this is attributable to the difficulty in passing the guide‐wire through the area of tight stenosis in CTO.^32^ In our analysis too, we found an overall lower success rate of CTO‐PCI with 70.4% in the elderly group and 78.3% in the nonelderly group. The success of CTO‐PCI was significantly reduced by around 37% in older adults. Answering the second question of our review, we found no significant reduction of long‐term mortality with successful PCI in the elderly by analyzing absolute events, however, pooled analysis of multivariable‐adjusted HR from limited studies did demonstrate that successful CTO‐PCI reduced the risk of long‐term mortality in the elderly. Furthermore, the risk of long‐term MACE was significantly reduced with successful PCI in both analyses. The results of our study concur with the outcomes reported with successful CTO‐PCI in the general population. Khan et al^30^ in a meta‐analysis of 23 observational studies have reported that successful recanalization of CTO lesions leads to a significantly reduced risk of all‐cause mortality (RR: 0.54 95% CI 0.45, 0.65) and MACE (RR: 0.70 95% CI 0.60, 0.83) as compared to failed PCI. It is known that around 99% of CTO lesions are less than 99% stenotic on histological examination.^3^ Furthermore, neovascularization takes place early with CTO and the budding capillaries provide minimal blood flow to the distal lumen keeping the myocardium alive.^3^ The restoration of antegrade blood flow via recanalization of CTO leads to the return of myocardial activity thereby improving clinical outcomes.^13^

Our review has some limitations. Foremost, our meta‐analysis included only observational studies and not randomized controlled trials. Selection bias may have skewed outcomes of the individual studies and thus influencing the results of this meta‐analysis. Second, there were significant baseline differences in the elderly and nonelderly groups. There was no reporting of multivariable‐adjusted HR for comparing outcomes of elderly and nonelderly groups. Several of these baseline variables could have generated bias in the results. Third, outcomes of CTO‐PCI also depend on the experience and skill of the operator. The influence of this variable could not be judged on the study results. Last, the number of patients in the elderly group in some studies was not high (<100 patients). This compounded by the limited number of studies available for each meta‐analysis may have reduced the statistical power of the review.

## 5 CONCLUSIONS

To conclude, our study indicates that elderly patients may have a tendency of higher in‐hospital MACE with significantly increased long‐term mortality and long‐term MACE after CTO‐PCI.

The success of PCI is significantly lower in the elderly versus nonelderly patients. In elderly patients with successful PCI, the risk of long‐term mortality and MACE is significantly reduced as compared to those with failed CTO‐PCI. Further larger studies using multivariable‐adjusted models are needed to strengthen the evidence.

## CONFLICT OF INTEREST

The authors declare that they have no competing interests.

## AUTHOR CONTRIBUTIONS

Chenmin Cui conceived and designed the study. Chenmin Cui and Zhichao Sheng collected the data and performed the literature search. Chenmin Cui was involved in the writing of the manuscript. All authors have read and approved the final manuscript.

## Supporting information


**FIGURE S1** Forest plot of in‐hospital major bleeding after CTO‐PCI in elderly versus nonelderly

**FIGURE S2** Forest plot of emergent CABG after CTO‐PCI in elderly versus nonelderly

**FIGURE S3** Forest plot of in‐hospital MI after CTO‐PCI in elderly versus nonelderly

**FIGURE S4** Forest plot of in‐hospital cardiac tamponade after CTO‐PCI in elderly versus nonelderly

**FIGURE S5** Forest plot of in‐hospital CVA after CTO‐PCI in elderly versus nonelderly

**FIGURE S6** Forest plot of success of CTO‐PCI in elderly versus nonelderly

**FIGURE S7** Forest plot of long‐term mortality after successful versus failed CTO‐PCI in elderly

**FIGURE S8** Forest plot of long‐term MACE after successful versus failed CTO‐PCI in elderly

**FIGURE S9** Forest plot of hazard ratios of long‐term mortality after successful versus failed CTO‐PCI in elderly

**FIGURE S10** Forest plot of hazard ratios of long‐term MACE after successful versus failed CTO‐PCI in elderly

**TABLE S1** Risk of bias in included studies
