# Supplementary figures and images for: Outcomes of percutaneous coronary intervention for chronic total occlusions in the elderly: A systematic review and meta‐analysis
Source: Clin Cardiol. 2020 Dec 17;44(1):27–35. doi: 10.1002/clc.23524 (PMC7803357; doi:10.1002/clc.23524)

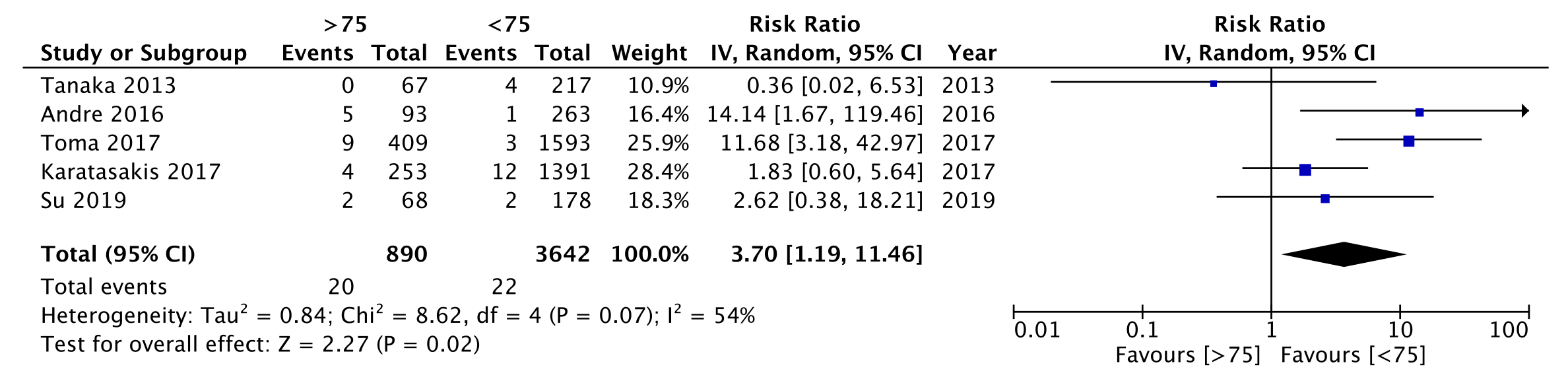

Supplement: Supplementary file 1 — FIGURE S1 Forest plot of in‐hospital major bleeding after CTO‐PCI in elderly versus nonelderly [file CLC-44-27-s001.tiff]

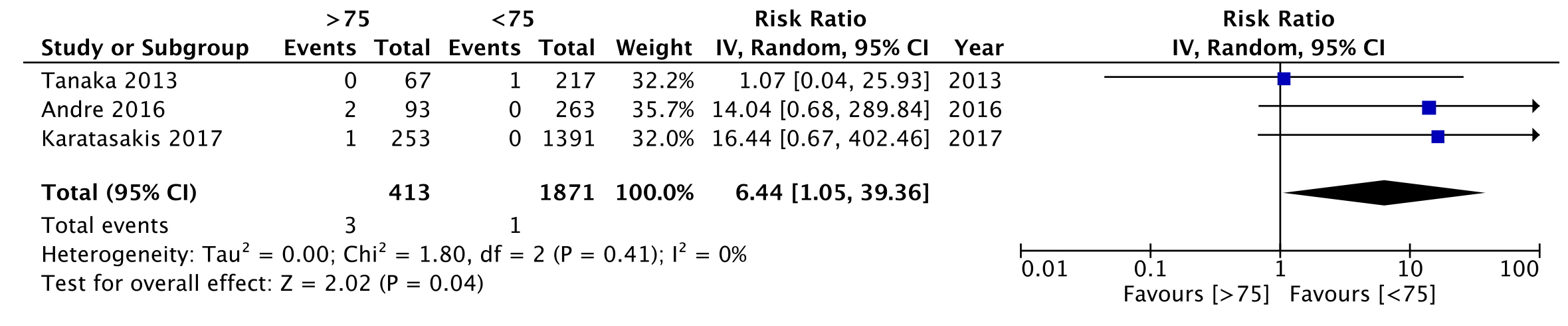

Supplement: Supplementary file 2 — FIGURE S2 Forest plot of emergent CABG after CTO‐PCI in elderly versus nonelderly [file CLC-44-27-s002.tiff]

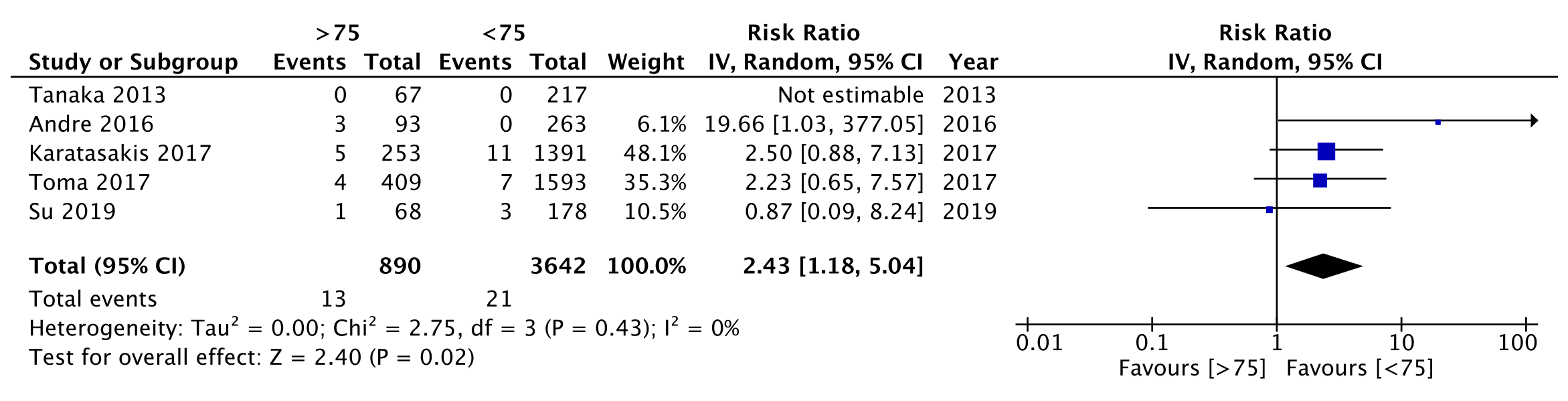

Supplement: Supplementary file 3 — FIGURE S3 Forest plot of in‐hospital MI after CTO‐PCI in elderly versus nonelderly [file CLC-44-27-s003.tiff]

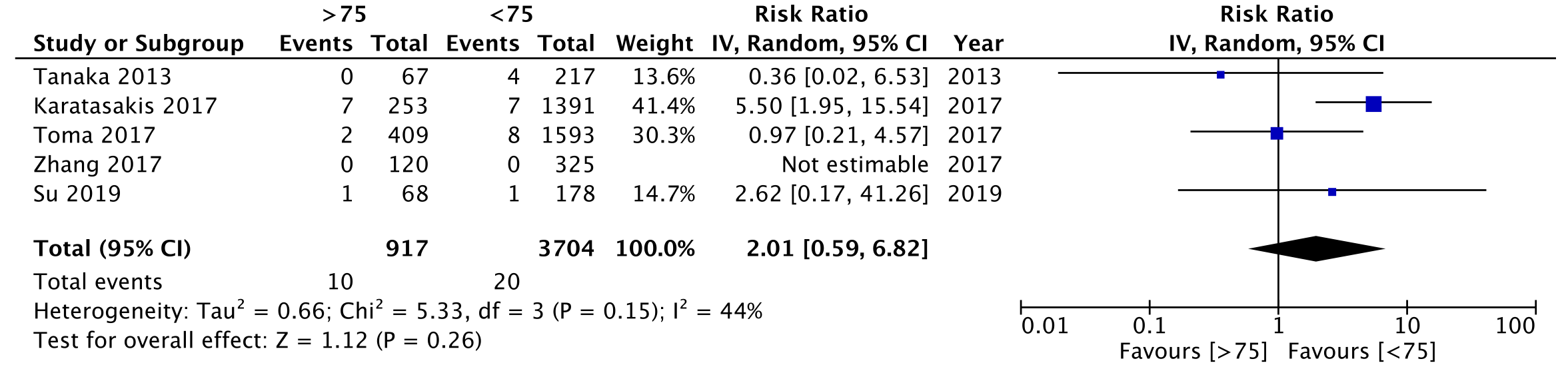

Supplement: Supplementary file 4 — FIGURE S4 Forest plot of in‐hospital cardiac tamponade after CTO‐PCI in elderly versus nonelderly [file CLC-44-27-s004.tiff]

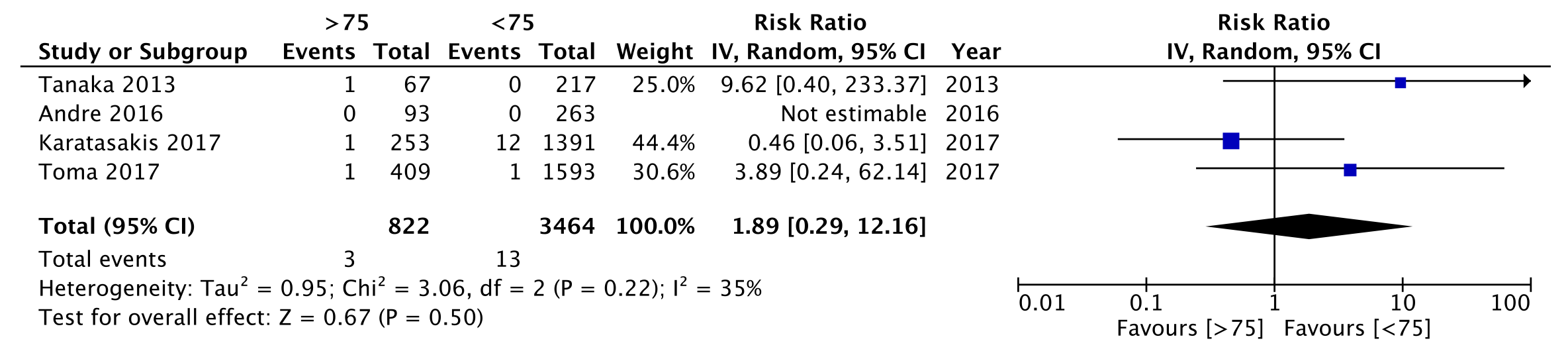

Supplement: Supplementary file 5 — FIGURE S5 Forest plot of in‐hospital CVA after CTO‐PCI in elderly versus nonelderly [file CLC-44-27-s005.tiff]

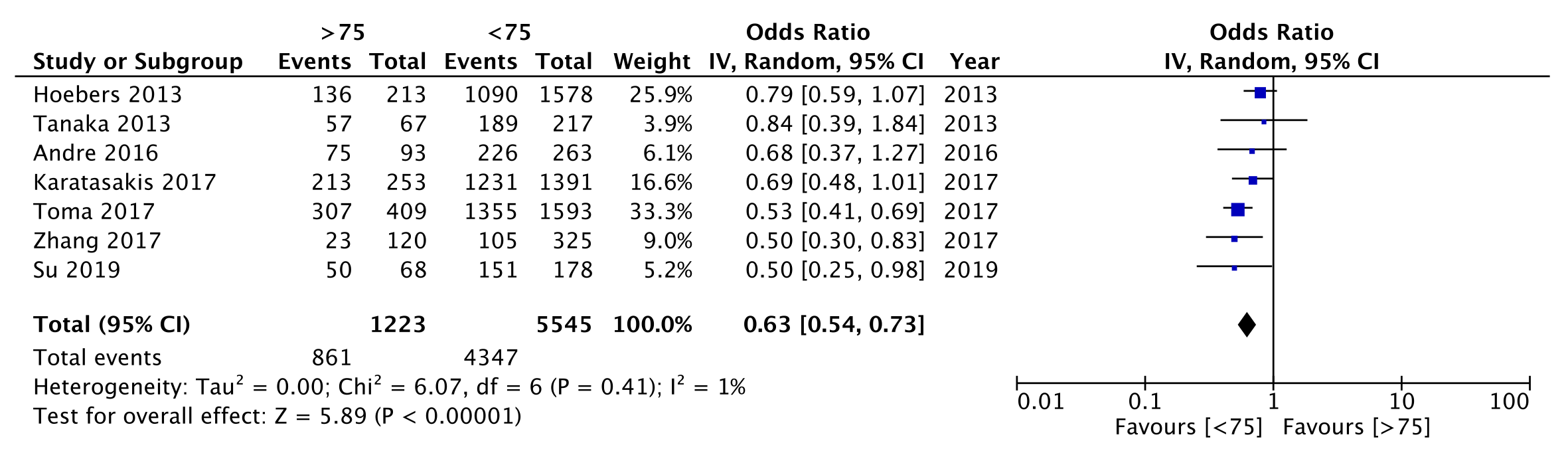

Supplement: Supplementary file 6 — FIGURE S6 Forest plot of success of CTO‐PCI in elderly versus nonelderly [file CLC-44-27-s006.tiff]

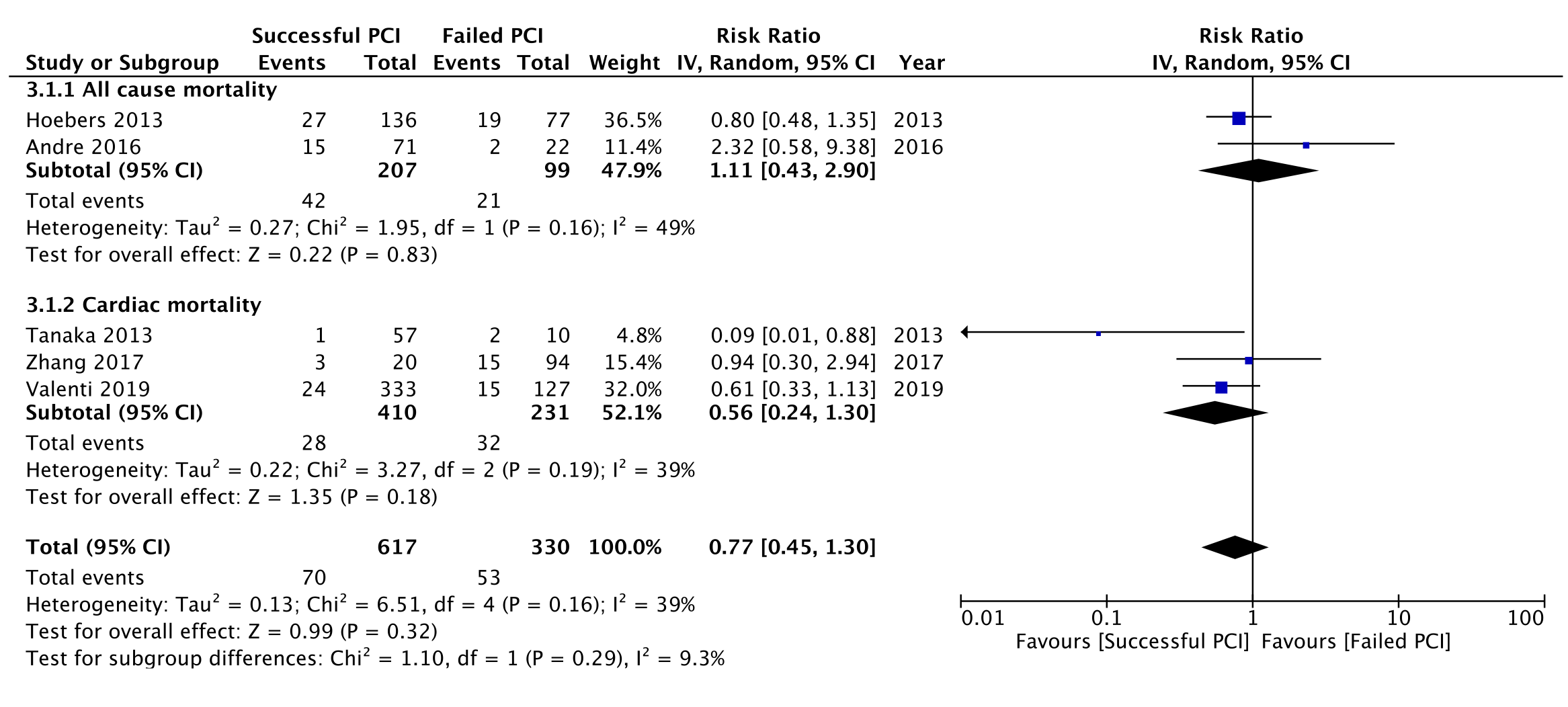

Supplement: Supplementary file 7 — FIGURE S7 Forest plot of long‐term mortality after successful versus failed CTO‐PCI in elderly [file CLC-44-27-s007.tiff]

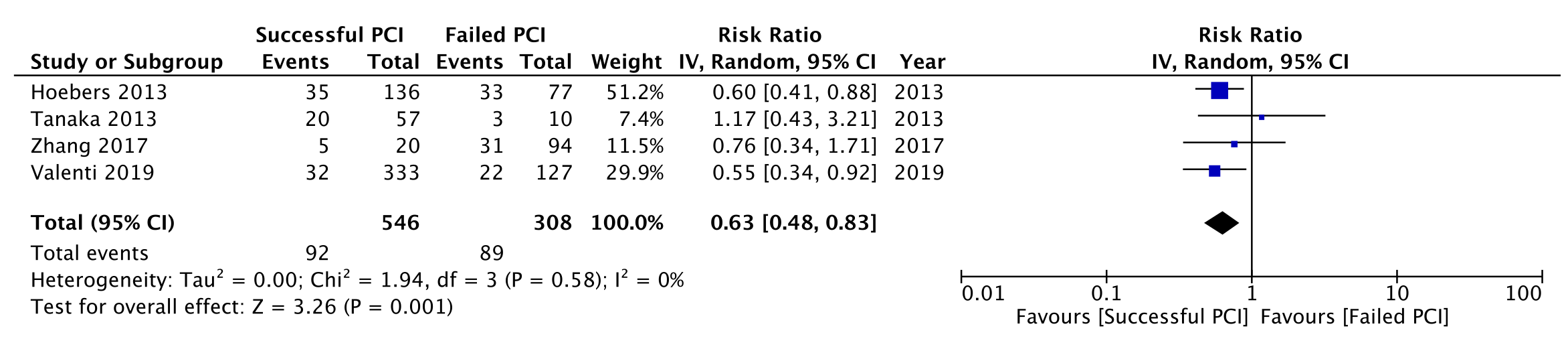

Supplement: Supplementary file 8 — FIGURE S8 Forest plot of long‐term MACE after successful versus failed CTO‐PCI in elderly [file CLC-44-27-s008.tiff]

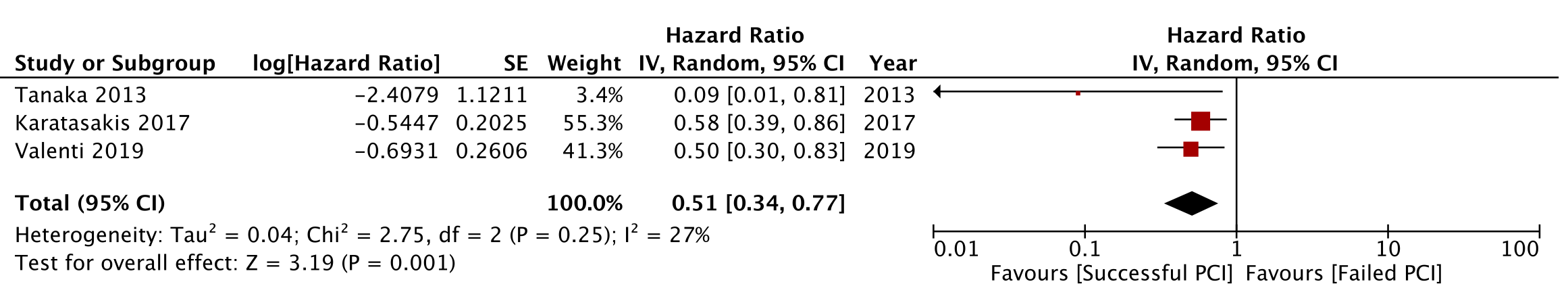

Supplement: Supplementary file 9 — FIGURE S9 Forest plot of hazard ratios of long‐term mortality after successful versus failed CTO‐PCI in elderly [file CLC-44-27-s009.tiff]

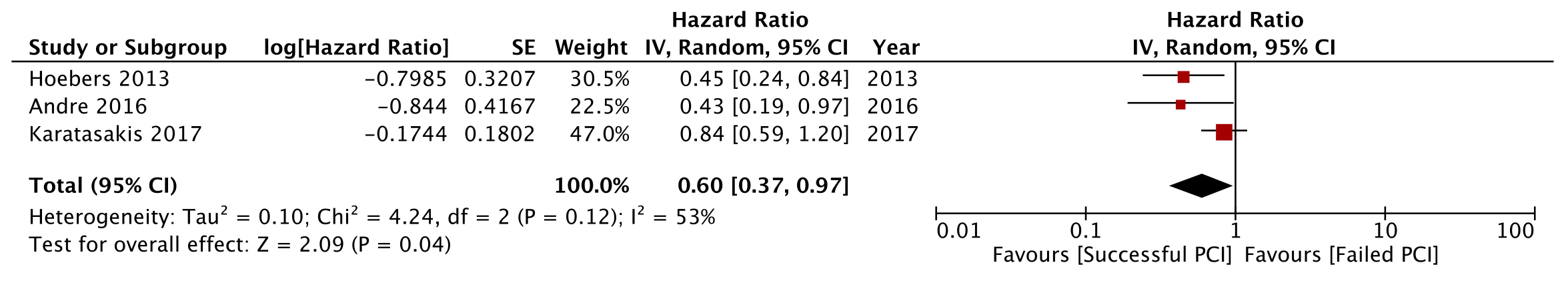

Supplement: Supplementary file 10 — FIGURE S10 Forest plot of hazard ratios of long‐term MACE after successful versus failed CTO‐PCI in elderly [file CLC-44-27-s010.tiff]
