# Supplementary material for: Outcomes of percutaneous coronary intervention for chronic total occlusions in the elderly: A systematic review and meta‐analysis
Source: Clin Cardiol. 2020 Dec 17;44(1):27–35. doi: 10.1002/clc.23524 (PMC7803357; doi:10.1002/clc.23524)
Supplement: Supplementary file 11 — TABLE S1 Risk of bias in included studies [file CLC-44-27-s011.docx]

Supplementary Table S1: Risk of bias in included studies

| Author/Year | Selection of participants | Confounding variables | Measurement of exposure | Blinding of outcome assessment | Incomplete outcome data | Selective outcome reporting |
| --- | --- | --- | --- | --- | --- | --- |
| Valenti et al^13^/2019 | High risk | High risk | Low risk | High risk | Low risk | Low risk |
| Su et al^14^/2019 | High risk | High risk | Low risk | High risk | Low risk | Low risk |
| Toma et al^16^/ 2017 | Low risk | High risk | Low risk | High risk | Low risk | Low risk |
| Karatasakis et al^17^/ 2017 | High risk | High risk | Low risk | High risk | Low risk | Low risk |
| Zhang et al^15^/ 2017 | Low risk | High risk | Low risk | High risk | Low risk | Low risk |
| Andre et al^18^/ 2016 | Low risk | High risk | Low risk | High risk | Low risk | Low risk |
| Tanaka et al^19^/ 2013 | Low risk | High risk | Low risk | High risk | Low risk | Low risk |
| Hoebers et al^20^/ 2013 | High risk | High risk | Low risk | High risk | Low risk | Low risk |
